# The Impact of Typhoon Haiyan on Health Staff: A Qualitative Study in Two Hospitals in Eastern Visayas, The Philippines

**DOI:** 10.3389/fpubh.2018.00208

**Published:** 2018-08-31

**Authors:** Julita Gil Cuesta, Joris A. F. van Loenhout, Maria L. de Lara-Banquesio, Juan M. Isiderio, Isabelle Aujoulat, Debarati Guha-Sapir

**Affiliations:** ^1^Centre for Research on the Epidemiology of Disasters, Institute of Health and Society, Université catholique de Louvain, Brussels, Belgium; ^2^Ormoc District Hospital, Ormoc, Philippines; ^3^Eastern Visayas Regional Medical Center, Tacloban, Philippines; ^4^Institute of Health and Society, Université catholique de Louvain, Brussels, Belgium

**Keywords:** disasters, health personnel, qualitative research, typhoon, floods

## Abstract

**Background and objective:** Understanding how natural disasters affect their victims is key to improve prevention and mitigation. Typhoon Haiyan strongly hit the Philippines in 2013. In Leyte, health staff of two hospitals had a key role as responders, but also as victims. Scarce literature is available on how health staff may be affected when being disasters' victims. We therefore aimed to understand Haiyan's impact for health staff at personal and work level.

**Methods:** We conducted semi-structured interviews in the two hospitals with doctors, nurses, midwives, watchmen and administrative staff in September 2016. We used a thematic analysis.

**Results:** The three main aspects reported as influencing staff were accessibility, safety and emotional aspects. Accessibility was a main difficulty, which prevented some staff from reaching the hospital, causing other staff staying longer on-call. Personal and family safety were affected, and due to remaining on-call immediately after Haiyan, staff members reported lack of information about their family situation. Faith was an emotional aspect repeatedly mentioned as a coping mechanism, and commitment to serve patients was for some respondents an essential argument to stay on duty.

**Conclusions:** Conflict between personal and professional concerns was present in health staff, making it difficult for them to prioritize work. Feeling unsafe was a common experience among health staff which influenced attendance to the hospital. Including temporary housing for staff and relatives close by the hospital can improve the extensive disaster risk during the typhoon season. In addition, established communication channels should be prioritized for staff on duty to find out about family members' wellbeing. We recommend faith and commitment to serve patients to be included in the preparedness programs in this setting.

## Background

Part of the destruction caused by natural disasters can be avoided (1), for example by having preparedness programs based on experiences from previous disasters. Understanding the way in which victims are affected during disasters is a prerequisite to improve prevention and mitigation, therefore preventing or reducing deaths and injuries during future disasters (1). This is especially relevant for hospitals, as they need to make an estimation of the expected impact of disasters, in terms of patient load. As such, hospital staff has a critical role in disaster preparedness and response.

On 8 November 2013, Typhoon Haiyan barreled through the islands of Central Philippines. It was one of the strongest typhoon on record and caused 7,986 deaths (2–4). In Leyte, the heaviest-hit island, the two major cities are Tacloban, which experienced severe damage from the storm surge and wind, and Ormoc, which suffered mostly wind and rain damage (5). According to the situation reports on 15 November 2013 (6), Eastern Visayas Regional Medical Centre (EVRMC) in Tacloban and Ormoc District hospital (ODH) in Ormoc City were the only two public hospitals still operational in the area. During and after Haiyan, health staff of these hospitals had an essential role and responsibility in patient care (7). Most staff members were themselves victims during Haiyan (8), which placed them in a double position as care providers and sufferers. This was therefore a unique opportunity to understand the conditions that influenced staff from EVRMC and ODH. The impact of disasters on hospital staff both at personal and at work level, has not been yet reported in-depth in the literature (9).

Our study aimed to gain insight in the impact at work and personal level of typhoon Haiyan for hospital staff. We therefore explored the conditions that influenced staff before and after Haiyan and the main aspects influencing those conditions.

## Materials and methods

### Study design

This is a qualitative descriptive study. A qualitative approach is useful when the topic of research is not widely known (10), with the aim of gaining richer understanding of processes and people's experience, as was the case for this disaster setting.

### Study population and sample procedure

We included two hospitals in our study, namely EVRMC in Tacloban and ODH in Ormoc. EVRMC is a tertiary hospital with 250 beds and ODH a secondary one with 100 beds capacity. We used a purposive sample of 12 health staff per hospital. We aimed to include in each of the hospitals, at least two persons of each of the following five professions categories to be interviewed: medical doctors (MD), nurses, midwives, watchmen, and administrative staff. For watchmen and maintenance all staff who fulfilled the criteria was included. The selection of the interviewees was carried out by the human resources managers of each of the hospitals, based on whether they were under contract in November 2013 when the typhoon occurred and were available for an interview during the data collection period.

### Data collection

We conducted semi-structured interviews (11) to collect information from health staff in the two hospitals, using an interview guide that we developed based on literature (Data Sheet 1). This guide contained open-ended questions which referred both to personal and work circumstances to each of the interviewees. We conducted interviews in the two hospitals from 19 September to 27 September, 2016. We carried out the interviews in a private room during working hours. No financial compensation was provided. The interviews lasted an average of 46 min (range 24–80 min). A team of two international researchers (JGC and JvL) and one translator, who was fluent in English, Tagalog and Cebuano conducted the interviews. Most interviews were conducted in English, although some participants preferred to have the interview in either Tagalog or Cebuano. All interviews were recorded.

### Data analysis

The interviews were transcribed ad verbatim from the digital recordings in the language that the interview had been performed in, to preserve the meaning of the interviewee's statements. The interviews conducted in Tagalog and Cebuano were then translated to English. We corrected the grammar in the quotes from the interviewees, when needed. We used conventional inductive content analysis as described in Elo et al. (10, 12). All data were analyzed based on the interview guide through open coding, creation of categories, and abstraction (12). We used thematic analysis (13, 14). A list of overarching themes was drafted and discussion between the two researchers interviewing (JGC, JvL) and a third researcher (IA) led to a consensus on themes. Following this, we explored possible relations between each of the themes. Related subthemes were added within the themes as they emerged in the data. Subthemes correspond to the results sections.

### Ethical considerations

Ethical approval was obtained from the ethics committee of EVRMC. Before the actual interview, the interviewer thoroughly explained the consent form and the study purpose to the participants, and gave participants time to read the consent form. We provided the consent forms in the participant's preferred language, either English, or one of the two local languages Waray Waray, Cebuano. All participants gave written consent before the interviews were conducted.

## Results

We present the results according to two sections: a first section where we describe the general conditions in these two hospitals during the first days according to the staff perceptions on hospital preparedness and response. A second section about the main conditions at work and personal level influencing staff performance, which was guided through the interview guide and where the different subsections emerged during the process of analysis.

We conducted 24 interviews in the two hospitals (12 in each). Thirteen were women and eleven were men (Table 1).

**Table 1:** Characteristics of health staff according to age, gender, profession, and presence at work during Typhoon Haiyan in two Hospitals in Eastern Visayas, the Philippines.

**Characteristics**		**Number (%)**
**GENDER**		
Male		11 (46)
**AGE CATEGORY**		
	25–40 years old	8 (33)
	41–55 years old	6 (25)
	above 5 years old	10 (41)
**PROFESSIONS**		
	Medical Doctor	6 (25)
	Nurse	7 (29)
	Nurse attendant	1 (4)
	Midwife	3 (12)
	Watchman and maintenance	3 (12)
	Administrative and laboratory	4 (16)
**PRESENCE IN THE HOSPITAL DURING TYPHOON HAIYAN**		
	Yes	11(47)
	No (back in 3 days or less after Haiyan)	7 (30)
	No (back in more than 3 days after Haiyan)	5 (21)

### Staff perception on hospital preparedness and response

The main perceived conditions influencing staff in the two hospitals before and after Haiyan follow the themes emerged during the interviews.

EVRMC and ODH are regularly affected by typhoons due to their location in Leyte, as reported by participants: “Based on statistics from previous years, there is an average of 24 typhoons that hit Region 8 or Tacloban per year. An average of two per month, so for us it's normal. (18, Maintenance engineer, HOSPITAL 1).” By 4 November 2013, when it was known that Typhoon Haiyan was arriving, the Philippine Government held emergency meetings for storm preparation in Tacloban (3). Similar meetings were held at hospital level: “On Monday before Haiyan, we had already a regular meeting with our disaster team (18, Maintenance engineer, HOSPITAL 1).”

#### Hospital and personal preparedness

Health staff reported different opinions on whether they considered their hospital and themselves prepared for Haiyan or not. Most of them thought there was not enough preparation: “Because most underestimated the strength of Haiyan and did not prepare much. (9, administrative, HOSPITAL 2).” “Yes, because I think the hospital also became complacent about it. If the hospital was prepared for this, I think there would have been more supplies. I think they would have provided a generator that was big enough, that could be used by the hospital. (1, nurse, HOSPITAL 2).” Only one respondent stated that he felt the hospital was properly prepared: “We had already regular meetings with our disaster team (before Haiyan). There was already coordination (in place), and we were actually prepared. (18, maintenance, HOSPITAL 1).” A recurrent point among the interviewees was that there was no awareness about the possibility of a storm surge “So, we were not informed, like for example about the storm surge. (11, nurse, HOSPITAL 2).”

#### Accessibility and attendance to the hospital

When Haiyan hit Tacloban and Ormoc in the early morning of Friday November 8th, some of the staff were at home and others were on duty at the hospital (see Table 1). At the hospital: “glasses were flying in all the directions… there was water everywhere … cars were floating (outside), because the water was indeed high.” (17, MD, HOSPITAL 1). Hours after the typhoon, there was no access through the roads, there were debris all over, roofs were blown off buildings, and the telephone network was hampered.

#### Transfer of the patients

In the immediate aftermath of the typhoon, the transfer of patients from one place of the hospital to another was repeatedly highlighted as an issue: “other staff had difficulty carrying the oxygen tanks, the babies, so maybe next time we should allocate an area to transfer the patients ahead. (4, MD, HOSPITAL 2).” “We should always be ready to evacuate the patients… during Haiyan, patients were brought in to the chapel to escape the water. (23, nurse, HOSPITAL 1).”

#### Availability of food, water and electricity

In addition, there was scarcity of food and water at both hospitals for patients and staff: “It was really difficult at that time, there was no food, no water. Everything was mixed up, in chaos. The personnel working would continue their shift because other personnel lived very far. (12, midwife, HOSPITAL 2),” “The problem was, we didn't have anything to eat. (24, MD, HOSPITAL 1).” In order to ensure enough meals for patients and staff, “lugaw” (rice porridge) was served in both hospitals: “Actually, during the time when we were on duty, we only had “lugaw.” (22, nurse, HOSPITAL 1).” External support started to arrive from local organizations after several days, in the form of generators and food donations: “On the fourth day, I saw different kinds of vests, from national and international organizations. (17, MD, HOSPITAL 1).” “It was flooded and there was lack of electricity because of the water there at the maintenance, I think it was around 2 meters high. Including our backup generator, flooded. So not operating. And luckily 4 days later, a team from the Department of Health already arrived, and brought a generator. (18, maintenance, HOSPITAL 1).”

#### Hospital functionality

Despite the situation, the two hospitals remained operational and received patients without interruption. Electricity was missing in both hospitals in the immediate aftermath. It was then reestablished at both Emergency Departments and missing for a few days at other departments. “During the first 2 days after the typhoon, there was no electricity for most of the wards. (10, MD, HOSPITAL 2).” “There was no light, only at the Emergency Room. (10, MD, HOSPITAL 2).” “When the doctor was operating at night, others who were helping had to hold the emergency flashlight. (5, security guard, HOSPITAL 2).” “We had no lights, we had nothing. (3, nurse, HOSPITAL 2).”

### Main conditions at work and personal level influencing staff performance

#### Accessibility to hospital

After the typhoon, most of the staff who had been working at the hospital on 8 November prolonged their shifts: “So I spent most of the time in the hospital until the following Monday (20, MD, HOSPITAL 1)” “…for me, even if my house was damaged and there was nothing repaired, I reported to duty. Without thinking about my own damaged house… (3, Nurse, HOSPITAL 2).” They stayed longer because they remained on duty until another colleague took over from them: “I'd been here for 2 days, since the next shift guard could not come. The roads were blocked by the trees. … (6, Security guard, HOSPITAL 2),” or even longer “Actually, I stayed here until the fifth day (17, MD, HOSPITAL 1).” Others went back home to assess the situation with their family members and belongings: “After my shift, I went home, it was my day off the following day. My house was devastated too, and my wife was angry cause she told me before not to report to work. She was left with my youngest daughter that time. The windows were forced open by the wind, the locks were broken down, the roof was blown away (19, nurse, HOSPITAL 1).”

For the staff who was not in the hospital during Haiyan, some did not report to work until days later: “I came back to work after 3 days, because I had to secure first my family. Then, after that, when my parents were already airlifted to Cebu, that's the time I had to report back (18, Maintenance, HOSPITAL 1).” The access to the hospital was an issue for most of the staff who were not on duty when the typhoon hit and had to reach the hospital after it: “The transportation was difficult, especially to the doctors coming from Tacloban and Palo, they could not go on duty (4, MD, HOSPITAL 2).” In the need of a particular professional, the hospital arranged their pick-up at home: “So our ambulance went to our house and told me, you should be on duty, because the doctor that was supposed to be on duty the following days, she could not come due to difficulty in coming here (24, MD, HOSPITAL 1).” In addition, staff looked for other means of transport to reach the hospital “So I tried to go outside. Most of the roads were impossible, a lot of debris etcetera. A lot of things covering the streets, you could not pass it. So what I did was get my mountain bike. I rode it, and every time there was an obstacle I just lifted the bike (24, MD, HOSPITAL 1).”

#### Safety

Participants mentioned safety as a key aspect in their daily life during the days after the typhoon: “We were afraid. We slept at night, we had a knife for protection, each one of us, each member of the family (23, nurse, HOSPITAL 1).” For some, it influenced their attendance to the hospital “And then I was not able to report again because my daughters wanted us to go to Manila because of the news that there were robbers. So we opted to leave… (24, nurse, HOSPITAL 1).”

#### Uncertainty/lack of information

Most of the staff who remained on duty reported suffering from the lack of information about what had happened to their relatives during Haiyan and the status of their belongings: “Inside the department, we actually had no idea on what was happening outside (19, nurse, HOSPITAL 1).” This uncertainty lasted up until 2 days for several staff members: “(The nurses from Tacloban working at HOSPITAL 2) didn't know the situation there (in Tacloban) until they came to their place, until they, themselves went to Tacloban (3, nurse, HOSPITAL 2).” “So, after 2 days, I went home to see my family at the next town. Because the roads were passable already. So, during Haiyan, we helped the patients but we were also thinking about our family, what's going (on) there (6, security guard, HOSPITAL 2).” Other staff members managed to verify whether their relatives were alive and fine, either by phone or by leaving the hospital: “Doctors were worried about their families. After they were sure that their families were ok, they returned to the hospital (20, MD, HOSPITAL 1).”

#### Faith

Staff's faith was an aspect repeatedly reported as important during the experience “We could not avoid (what was happening), all we could do was to pray (15, administrative, HOSPITAL 1).” “We had no food, but we still thanked God for that experience. Even if our house was destroyed (8, midwife, HOSPITAL 2).” Praying was a spontaneous response for some of the staff in the first hours and days: “then I went inside the obstetrics doctors quarters and we prayed the rosary… I cannot imagine how I managed to do it. And those many patients, there were none who died. With God's grace. (17, MD, HOSPITAL 1).” “At 11 o'clock (on November 8th) we decided to go downstairs to pray to the lord. We met a group of other survivors that are Christians too, and they joined us in prayers. Whatever happened, it was his will. So all we can do is to pray to God. We prayed and God heard, the wind got smaller (15, administrative, HOSPITAL 1).”

#### Commitment to serve patients

Several of the respondents reported their motivations to either stay on duty during the typhoon or to return early to the hospital. Commitment to serving patients was perceived as a key issue in this decision: “What it is in our mind is commitment and dedication to help people. Because for me, even if my house was damaged, and there was nothing repaired, I reported to duty. Without thinking about my own damaged house. We become selfless. We don't think any longer about ourselves, but about who are the bigger victims (3, nurse, HOSPITAL 2).” This commitment was described as a support in the aftermath: “there were times that I could not avoid to think of the situation in the house or to the children at home. But since I had a responsibility here in the hospital and to the patients, I would try my best to focus and concentrate on working (7, nurse, HOSPITAL 2).”

#### Personal belongings affected

It was repeatedly stated that health staff had their personal belongings affected by the typhoon, most frequently the roof of their house: “Not even a single roofing left. (12, midwife, HOSPITAL 2).” Other times, they were not able to stay at their own house “For the first 3 days, my family stayed here in the hospital. But then, after that, I built a small hut, like a temporary shelter, for the family, so they really didn't have to stay in the evacuation center (5, security guard, HOSPITAL 2).”

## Discussion

This is one of the first studies that looked at the response of hospital staff after a major typhoon. We found that local staff coped with a double burden during and after Haiyan. The main aspects reported as influencing staff conditions affected them at personal and at work level. Accessibility to hospital, their own safety and lack of information about the situation of their relatives affected them at the two levels. Faith and their commitment to treating patients were highlighted as emotional aspects that influenced them.

This dual challenge at work and personal level faced by health care staff has been described after other disasters, like the 7.1 magnitude earthquake in 2010 in New Zealand (15). Due to a double request for attention, most staff members felt a dilemma between prioritizing their personal life, including family members and personal belongings, or their work during the hours and days following Haiyan. This is similar to hospital staff in other disasters, where they felt the responsibility to provide the best care possible, even if they were affected themselves (16). A first group of respondents in our study reported to have ensured their personal side first. Secondary stressors such as housing difficulties and loss of social networks have been described to affect staff's decision in this (16), factors that were confirmed by our findings. A second group prioritized their attendance to work. Participants reflected how commitment to serve the patients worked as a motivation. Differences in terms of motivation were anecdotally observed between the medical staff (MD, nurses, midwives) and the other professions interviewed (laboratory, watchmen, clerk, and registry staff). Health staff, especially if more senior, tended to prioritize more their work and emphasized their motivation to serve patients. Similarly, differences had also been observed in similar settings between nurses and general practitioners, probably explained by professional cultural differences (15). This difference in perceived responsibility between different staff profiles should therefore be considered when preparing for a disaster, since drivers to work are different for different professions. The influence of income on behaviors and choices in stress situations is an important factor. There is indeed little research done on this question and it needs further examination to improve preparedness. The issue deserves in-depth analyses of how income levels affect response in catastrophic settings.

Major challenges for hospital staff consisted of accessing the hospital to work and their own houses. The difficulties in accessing the hospital have also been described in other typhoons (17). In order to improve staff reporting in disaster settings like Leyte, which suffers from repeated typhoons, preparedness and response plans should contain sections on the availability of staff in the hospital or close surroundings beforehand to ensure the presence during and directly after the typhoon. Other hospitals have spontaneously provided shelter to staff relatives and the general community in the aftermath of disasters like Katrina (17). Such a measure could help in ensuring that health staff on duty can consider their family situation under control. Thus, hopefully avoiding them the need to prioritize between family safety and work attendance.

Safety needs were perceived as important, and in some circumstances it was reported to be the reason to have left Leyte. Safety is fundamental in the hierarchy needs (18), and especially after disasters, the perception of absence of danger becomes key (19).

Lack of information about the family, personal belongings and general context was pointed out as a factor. This uncertainty has also been reported as a stressor in other disasters. In order to improve the staff's knowledge about the external context, communication lines should be established, as to ensure that the staff would be updated about their relatives, and that the information received by the hospital is well distributed.

Faith was reported by health staff as an aspect of their coping behavior. In a focus group study after Haiyan, faith showed to have a vital role in building resilience in the community (20). During our study, the acceptance of the disaster situation was linked to faith in health staff. Faith was also expressed to contribute to the motivation to serve patients in one participant. During the aftermath, spontaneous praying occurred in the first 48 h inside the hospital and more organized praying ceremonies in the months after. In the Haiyan aftermath, prayer was an unanticipated central element of resilience in the community (20). Evidence suggests that rituals can structure the experiences of loss in the aftermath of a disaster, give meaning to the traumatic event and help the community cope with their suffering (21). To our knowledge, no previous studies have described this aspect in the disaster aftermath in health staff. This is important in the Philippines setting, where moments that allow praying should be part of the preparedness and early response at hospital level, and for a next disaster, time and space could be offered to hospital staff to engage in praying. This should be incorporated in preparedness and response plans.

## Limitations

The main limitation that we faced was a recall bias, since the interviews were undertaken almost three years after the typhoon. Still, most likely due to the magnitude of the event, staff was vividly reporting about it. In addition, there may have been a selection bias, since hospital staff was directly selected by the hospital human resources managers. When conducting the interviews, different cultural background between the researchers and the interviewees may have influenced the interpretation of some messages. The presence of a local translator, who was herself present during Haiyan may partly have prevented this bias from occurring.

## Conclusions

Hospital staff reported the need to prioritize between their personal and work circumstances in the aftermath of Haiyan. This double reality should be integrated in the hospital preparedness plans. Ways to improve staff attendance and motivation should also be incorporated. Feeling unsafe was a common experience among health staff which influenced attendance to the hospital. Safety of staff and their relatives should be ensured, which may include housing for staff and relatives within the hospital premises. External factors, such as access to the hospital and lack of information, influenced the staff working conditions as well. Established communication lines should be put in place to increase availability of information, such as radio communication. Faith was perceived as an important coping mechanism in this setting. Commitment to serving patients was perceived as a key motivation in the aftermath. We recommend faith and commitment aspects to be considered in the preparedness plans.

## Author contributions

JG, JvL, and DG-S conceived the study. JG, JvL, MdL-B, and JI collected and revised the data. JG analyzed the data. JG, JvL, IA, and DG-S interpreted the data. JG wrote the manuscript. All authors revised the manuscript, and approved the final version.

### Conflict of interest statement

The authors declare that the research was conducted in the absence of any commercial or financial relationships that could be construed as a potential conflict of interest. The reviewer MFDA and the handling Editor declared their shared affiliation.

## Abbreviations

EVRMC: Eastern Visayas Regional Medical Center
ODH: Ormoc District Hospital.
