# Supplementary material for: The Impact of Typhoon Haiyan on Health Staff: A Qualitative Study in Two Hospitals in Eastern Visayas, The Philippines
Source: Front Public Health. 2018 Aug 31;6:208. doi: 10.3389/fpubh.2018.00208 (PMC6128081; doi:10.3389/fpubh.2018.00208)
Supplement: Supplementary file 1 [file Data_Sheet_1.doc]

**Annex 1. Interview guide to health staff**

Introduction

We are public health researchers working in Belgium, we came one year ago to see which were the main issues/worries from health staff after Haiyan. Based on our first visit, we understood that it would be important to get an insight/better understanding of the impact of Haiyan at personal and work level.

We would like to ask you questions related to your personal experience and your professional life after Haiyan. We would like to record the interview if that is fine with you, which will only be used to help us to remember all what you said.

Please read carefully the following consent form, and if you agree, you can sign and we can start with the interview.

Feel free to share any related idea that you may find important. Let’s start.

1. Socio-demographic information
   - Age
   - Gender
   - From when to when did you work in the hospital?
   - What kind of job did you start with in the hospital, and which changes have taken place?
2. Personal circumstances

- Can you tell us about how typhoon Haiyan affected you on a personal level, or how it affected your life?

1. Work-related circumstances

- What was it like when you went back to work?
  - How did you perceive your ability to work after Haiyan?
  - What were the personal working conditions like?
  - How did your situation at home affect your ability to work …?
  - How do you think the quality of care was influenced in your department/hospital, just after Haiyan? And in the three months following Haiyan?
  - Based on your experience, what could be done differently for the next typhoon in terms of work?
